# Seeing Skin Tone Differently: An Experimental Comparison of Objective Measurements and Subjective Perceptions

**DOI:** 10.3390/bioengineering13080919

**Published:** 2026-08-13

**Authors:** Jinani Sooriyaarachchi, Catherine Proulx, Linda Pecora, David Rivest-Hénault, Thomas Vaughan, Marc-André Rainville, Aidan Saull, Annie Fortin, Di Jiang

**Affiliations:** Medical Devices Research Centre, National Research Council of Canada, Boucherville, QC J4B 6Y4, Canada

**Keywords:** skin tone, skin tone classification, skin hue, colorimeter, individual typology angle, melanin index, palette, Fitzpatrick skin type, skin tone measurement

## Abstract

Human skin tone is an important consideration in pulse oximetry and evaluating skin health conditions. Various tools are available to measure skin tone, including visual scales, palette based estimations, and optical sensor based devices. The objective of this experimental study is to compare common skin tone measurement methods. We used Fitzpatrick Skin Type (self- and observer-reported), Pantone SkinTone Guide, Delfin SkinColorCatch, and Nix Spectro 2 devices to measure the forehead skin tone of 52 participants. We compared measurements in L*a*b color space and in individual typology angles (ITA). We observed statistically significant correlation between methods, including in specific color space components (L* and ITA). Between the device measurements, we observed discrepancies in ITA values. Such discrepancies, together with a lack of standard or ground truth method, show a need for further research into skin tone measurement. We suggest in the interim that research and clinical work adopt protocols that take current methodological limitations into consideration, for example through the parallel use of different methods if applicable. Further work is needed to develop validated, reliable and consistent skin tone measurement tools.

## 1. Introduction

Skin tone is an important consideration in many medical applications, including skin reconstruction [1], monitoring skin health conditions such as skin cancer [2], and pulse oximetry [3]. To elaborate on one example, darker skin tones have been associated with reduced device accuracy in pulse oximetry, prompting efforts to incorporate skin tone measurements to improve the underlying modeling, calibration, and reporting for training data [3]. A skin tone characterization methodology for the international standard ISO 80601-2-61:2026 (Ed 3) on pulse oximeter basic safety and essential performance defines the expectation for balanced participant enrollment across the world’s range of skin tones [4]. Contactless pulse oximetry [5] (e.g., with a color camera) is an emerging modality that introduces additional complexity due to interactions between skin color and the surrounding environment, many of which remain poorly understood. Skin tone bias in medical devices is likely due to systemic factors that lead to insufficient validation across diverse skin tones [6]. Continued research and technological development involving skin tone relies on the use of appropriate representations and accurate skin tone measurement tools, as well as a clear understanding of their limitations in practical applications.

Various scales and methods for measuring skin tones have been proposed over the years, and research in this area continues to evolve. Prior studies have compared some of these scales and tools, typically focusing on a limited number of methods or pairwise comparisons, with few comprehensive cross-method evaluations. A typical finding is that subjectively-reported skin tone shows variability both between observers and when compared to a commercial color measurement device. Although devices are often considered objective and more consistent compared to subjective reporting, few studies have directly compared skin tone reported by different devices for the same individual.

The objective of this study is to show how measurements compare between methods, and to highlight current need in the state of the art. To this end, we present a comprehensive analysis of skin tone data from 52 study participants, measured using multiple approaches: self-reported measurements, observer-reported measurements, palette-based measurements, and objective measurements from two commercially-available devices. The findings offer insights for researchers in choosing appropriate skin tone measurement tools. To our knowledge, this work is the first to directly compare the specific combination of skin tone scales and measurement devices examined in this study.

## 2. Background

Skin tone, as visible to humans or devices is the result of light-absorbing chromophore compounds in the skin, most prominently melanin, oxy-hemoglobin, and deoxy-hemoglobin [6,7]. Various methods have been proposed to objectively describe skin tone, including numerical scales, color charts, and multi-component color spaces. In general, methods to measure skin tone fall into one of three categories: text-based, palette-based, or optical-sensor-based.

- Text-based methods

The most prominent text-based method is the Fitzpatrick Skin Type (FST) classification scale, which ranges from I to VI and may be either self-reported or observer-reported. It was originally developed to determine doses of UV light for phototherapy and is based on how an individual’s skin reacts to sunlight [8]. A more recent development is the Monk Skin Tone (MST) Scale, a 10-tone classification system developed by Monk et al. [9] in collaboration with Google Research. It was designed to provide a more inclusive representation of human skin tones than traditional scales such as the Fitzpatrick scale, with the goal of improving fairness and diversity in technology and research applications. Despite its limited accuracy and precision relative to objective measures [6,10,11], especially for darker-skinned individuals [10], the FST scale remains widely available and commonly used [11]. Although easy to use, FST does depend on a reporter’s (subjective) perception.

b. Palette-based or image-based methods

By presenting a set of color palettes or images, skin tone can be self-reported or observer-reported. The Pantone SkinTone Guide offers 138 swatches representing a diverse set of skin tones, to be compared visually with an individual’s skin tone [12]. This method is relatively low-cost, and grants more precision than FST, though it can be more challenging to use consistently and properly [6]. Similarly to FST, these methods depend on a reporter’s (subjective) perception.

c. Optical -sensor -based methods

Skin tone can be measured objectively using purpose-built colorimeters or spectrophotometers. Such devices include the Delfin SkinColorCatch (Delfin Technologies, Kuopio, Finland) [13], the Nix Pro 2 [14,15], and the Nix Spectro 2 (Nix Sensor Ltd., Hamilton, ON, Canada) [16]. The Delfin is a colorimeter that has been used for skin tone measurements in multiple studies [3,6,17,18,19]. The Nix Spectro 2 is a spectrophotometer that has been used by a team of plastic and reconstructive surgeons to improve skin tone matching in face transplantation procedures [1]. Such devices allow protocols that may be less prone to inter-observer variability, but they require training for proper application (e.g., sampling location, pressure applied, calibration, and environmental conditions).

Values reported by colorimeters or spectrophotometers may include a color space representation such as CIELAB, i.e., $L^{*}a^{*}b^{*}$. Another representation could be the Individual Typology Angle (ITA), which is a classification based on precise quantification of color parameters [20]. The ITA offers an objective measure of skin tone based on the L* (lightness) and b* (yellowness-blueness) values derived from CIELAB color space.

### Previous Comparison Studies

Comparisons among the aforementioned skin tone measurement methods have been explored in multiple research studies.

Weir et al. [6] reviewed methods and representations of skin tone, highlighting the absence of a universally accepted gold standard, and a general lack of validation across approaches. They also emphasized the presence of systemic biases, particularly affecting individuals with darker skin tones. They call for greater attention to device validation studies and a more critical evaluation of limitations in different skin tone measurement methods. We note from Weir et al. [6] that automated methods based on machine learning have been gaining interest from the research community. These may operate on images, for example, and classify or estimate skin tone. As per authors, these automated methods can have similar limitations as the manual methods on which the training data is based. Thus the focus of the current work remains on manual methods.

Cook et al. [21] showed that human assessments of facial skin tone can be error prone due to multiple factors including individual differences in color perception, the presence of surrounding colors, and the influence of perceived race. Lipnick et al. [18] recently conducted a study involving three subjective skin tone scales (Fitzpatrick scale, Monk Skin Tone and Von Luschan) and two objective devices (Konica Minolta CM-700d spectrophotometer (Konica Minolta Inc., Tokyo, Japan) and the Delfin SkinColorCatch colorimeter (Delfin Technologies, Kuopio, Finland)). Notably, they found that there were significant discrepancies between different measures when using subjective scales and race (as defined by the United States National Institutes for Health) to determine skin tone. Similarly, Verkruysse et al. [22] acknowledged the need to distinguish two complementary types of skin tone assessments: subjective and objective skin pigmentation measurement methods. They showed that the skin color perceived and assessed by humans using skin tone scales (Pantone Skin Tone, Fitzpatrick scale, and Von Luschan) can be substantially different from skin color measured objectively using colorimeters (Konica Minolta CM700d).

The remainder of this paper is structured as follows: Section 3 outlines the methodology, detailing the data collection and skin tone measurement procedures. Section 4 presents the results of the comparative analysis, followed by discussions of these findings in relation to prior work and practical implications in Section 5. Section 6 concludes the paper and suggests directions for future research.

## 3. Methodology

### 3.1. Study

Study participants were recruited in two sequential phases. In the first phase, prospective participants were asked to provide their best estimate of their Fitzpatrick Skin Type (FST). This information was used to guide participant selection and ensure balanced representation across skin tones. A second phase of targeted recruitment was conducted by specifically inviting individuals with light or dark skin (e.g., FST I, II, V, and VI), as these skin types were more difficult to obtain in balanced sample populations. This two-phase approach allowed the creation of a more comprehensive and balanced dataset.

A total of 52 participants (27 male and 25 female), aged 21 to 71 years, provided informed consent to participate in the study. The experimental protocol was reviewed and approved by the National Research Council of Canada Research Ethics Board (Protocol No. 2024-40), and all experiments were conducted at two sites by three trained experimenters.

**Figure 1:**
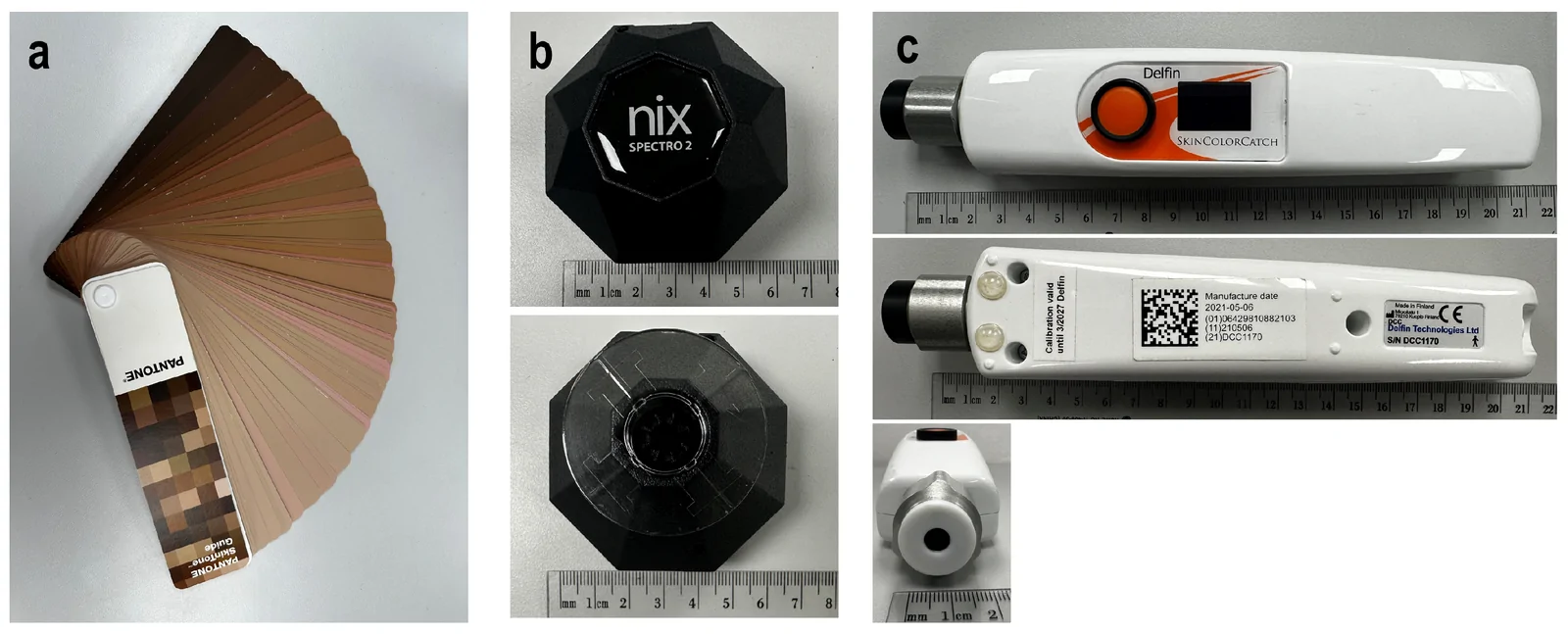
Palette-based and commercially-available skin tone measurement devices used in this study: (**a**) Pantone skintone guide, (**b**) Nix Spectro 2 spectrophotometer (top and bottom (with aperture) view), (**c**) Delfin SkinColorCatch colorimeter (front, back, and aperture view).

Skin tone measurement methods representing all three categories described in the Section 2 were included in this study. Five distinct methods were used to measure participants’ skin tone:

1. Self-reported FST.
2. Observer-reported FST, as assessed by the experimenter.
3. Pantone SkinTone Guide [12] (hereafter referred to as Pantone), for which color code was determined jointly by the participant and the experimenter (Figure 1a).
4. Nix Spectro 2 spectrophotometer (Nix Sensor Ltd., Hamilton, ON, Canada) (hereafter referred to as Nix) [16], from which $L^{*}a^{*}b^{*}$ values were used for analysis (Figure 1b).
5. Delfin SkinColorCatch colorimeter (Delfin Technologies, Kuopio, Finland) (hereafter referred to as Delfin) [13], from which $L^{*}a^{*}b^{*}$, ITA, MI (melanin index; quantitative measure of skin pigmentation), and EI (erythema index; quantitative measure of skin redness) values were used for analysis (Figure 1c).

When a participant or experimenter could not decide between two FST, they were instructed to indicate a half level (e.g., 1.5, 2.5, etc.). Both the Nix and Delfin devices were calibrated at the start of each day to ensure measurement accuracy. Skin tone measurements were collected from the central region of the forehead, just above the eyebrows, in accordance with the FDA’s recently released draft guideline document [23]. Two consecutive readings were taken at this location to ensure consistency, and the average of these measurements was used for all subsequent analyses.

### 3.2. Data Analysis

To assess the reliability and consistency of the skin tone measurement devices (Delfin and Nix) across repeated measurements, the Intraclass Correlation Coefficient (ICC) was calculated. ICC values range from 0 (no agreement) to 1 (perfect agreement).

To evaluate the agreement between measurements obtained using different methods, we used common representations for skin tone. One of these was the ITA, because of its widespread use in both clinical and research contexts. Delfin provides ITA values directly. Nix measurements were in the $L^{*}a^{*}b^{*}$ space, and were subsequently converted to ITA using (1) [20]. Pantone codes were converted first to RGB (red, green, blue) format using the Pantone Connect app [24], then transformed to $L^{*}a^{*}b^{*}$ and subsequently to ITA values. Because there is no direct conversion from FST to ITA values, FST values were mapped to skin tone range based on ITA boundaries defined in [13,25], as shown in Table 1.

(1) $$\mathrm{ITA}{}^{\circ}=\frac{180}{\pi }\arctan\left(\frac{L^{*}-50}{b^{*}}\right)$$

To help explain nuanced differences and relationships involving skin tone, we generated additional scatterplots and performed Pearson correlations involving other values. Among these values were the MI and EI as reported by the Delfin.

Since ITA incorporates only the $L^{*}$ and $b^{*}$ components of the CIELAB color space, we also calculated a metric that accounts for all components of the $L^{*}a^{*}b^{*}$ space and therefore covers a broader range of hue. This metric, denoted as $\Delta^{*}$, quantifies the color difference between two points $x_{1}$, $x_{2}$ in $L^{*}a^{*}b^{*}$ and is computed using (2).

(2) $$\Delta^{*}=\sqrt{{\left(L_{1}^{*}-L_{2}^{*}\right)}^{2}+{\left(a_{1}^{*}-a_{2}^{*}\right)}^{2}+{\left(b_{1}^{*}-b_{2}^{*}\right)}^{2}}$$

#### Statistical Analysis

For determining agreement involving ITA and $L^{*}a^{*}b^{*}$ components between different skin tone measurement methods, scatterplots were generated and Pearson correlation (r) was computed along with it’s statistical significance ($p_{r}$).

For testing statistical significance in observed differences, we used non-parametric tests that did not assume normality. For unpaired data, the Mann-Whitney U-Test was applied with *p*-value reported as ($p_{u}$). For paired data, the Wilcoxon Signed Rank Test was used with the *p*-value reported as ($p_{w}$).

## 4. Results

Skin tone measurements from a total of 52 participants were collected and analyzed in this study. Varied skin tones were represented in this data as shown in Table 1.

### 4.1. Reliability of Repeated Measurements

Both devices showed reliability in repeated measurements of skin tone as quantified using the ICC (0.989 (CI [0.98, 0.99]) for Delfin and 0.966 (CI [0.94, 0.98]) for Nix).

### 4.2. Comparison of ITA Values Across the Study Population

**Figure 2:**
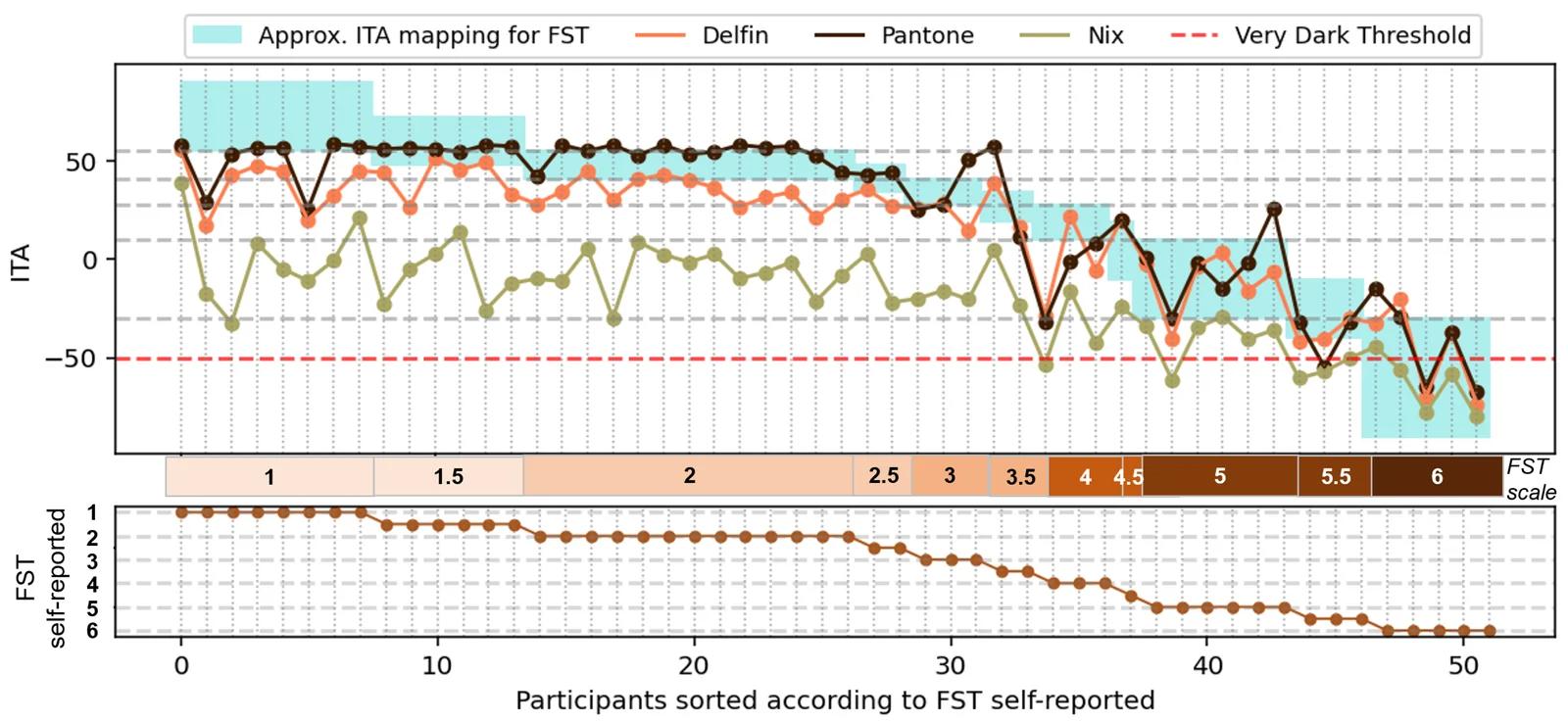
Comparison of ITA values from different measurement methods. The “very dark” threshold as per Lipnick et al. is shown [18].

ITA measurements from Delfin, Nix and Pantone were compared alongside FST self-reported skin tone values (Figure 2). Statistically significant correlation was observed between derived ITA from Pantone and Nix (r = 0.87, $p_{r}$ = 1.14 × 10^−16^), Delfin and Pantone (r = 0.95, $p_{r}$ = 2.96 × 10^−26^), and Delfin and Nix (r = 0.91, $p_{r}$ = 7.55 × 10^−21^).

The ITA values obtained using Nix (range: −20.71° ± 25.24°) were significantly lower than those obtained with Pantone (range: 25.69° ± 38.16°, $p_{w}$ = 3.50 × 10^−10^) and Delfin (range: 15.02° ± 32.62°, $p_{w}$ = 3.50 × 10^−10^). The Nix-derived ITA was lowest for every measurement (which is consistent with the $p_{w}$ values being identical for the two tests). The difference in ITA between Pantone and Delfin was also statistically significant ($p_{w}$ = 1.91 × 10^−6^).

We observed higher differences in ITA values between Delfin and Nix for participants with lighter skin tones (FST I-III determined using Delfin) compared to participants with darker skin tones (FST IV-VI) (Figure 2, Table 2).

### 4.3. Comparison of Palette-Based and Optical-Sensor-Based Measures with Text-Based Skin Tone

**Figure 3:**
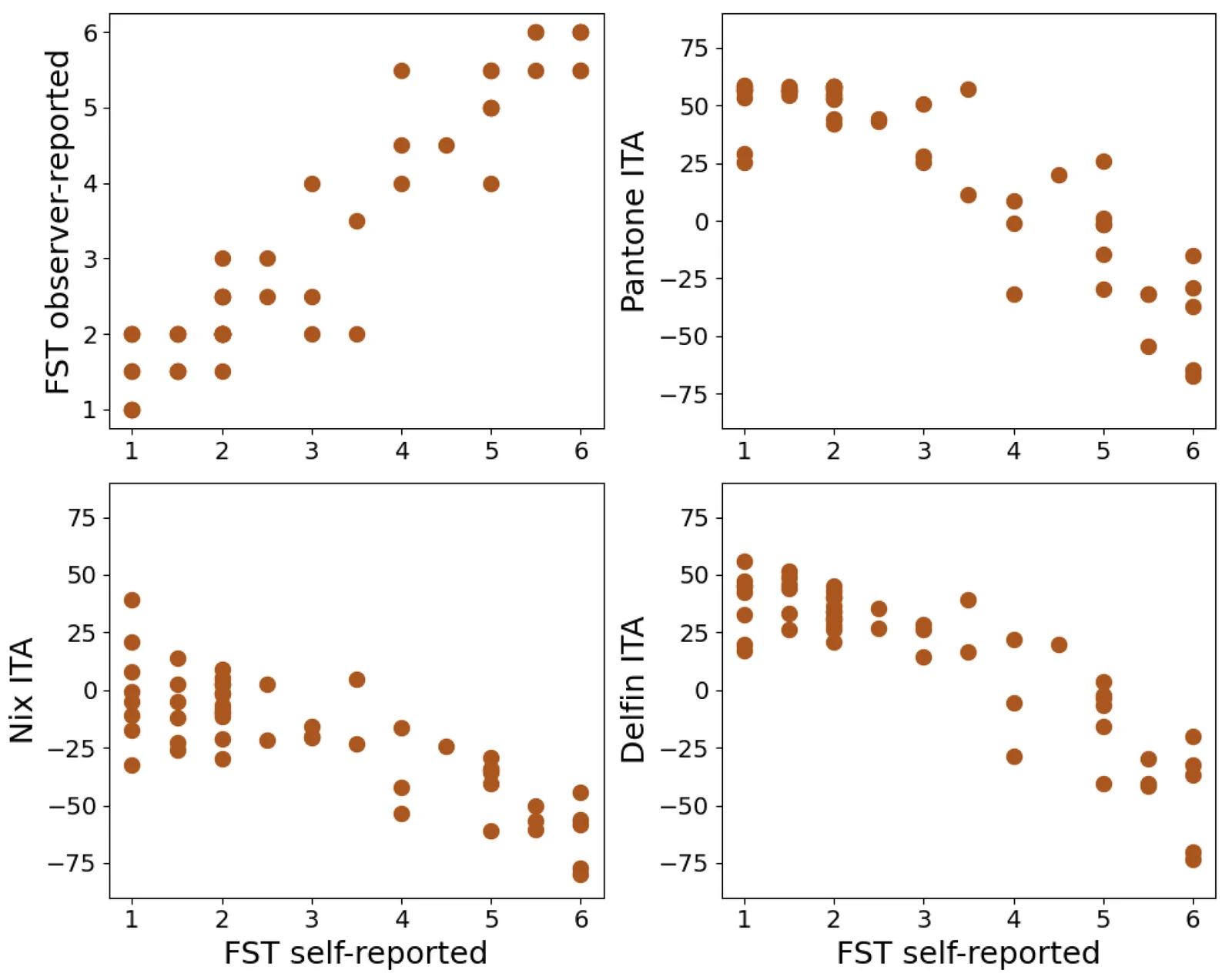
Fitzpatrick self-reported skin tone values compared with observer-reported values, Pantone-derived ITA, and spectrum based ITA values.

Using self-reported FST as a reference, we computed its correlation with other measures (observer-reported FST, Pantone, Nix-derived ITA, and Delfin ITA) in Figure 3.

We observed statistically significant correlations between self-reported and (1) observer-reported FST (r = 0.95, $p_{r}$ = 4.81 × 10^−27^); (2) Pantone-derived ITA (r = −0.89, $p_{r}$ = 5.99 × 10^−19^); (3) Nix-derived ITA (r = −0.82, $p_{r}$ = 1.74 × 10^−13^); and (4) Delfin ITA (r = −0.88, $p_{r}$ = 5.65 × 10^−18^).

### 4.4. Agreement in Pantone and Delfin

**Figure 4:**
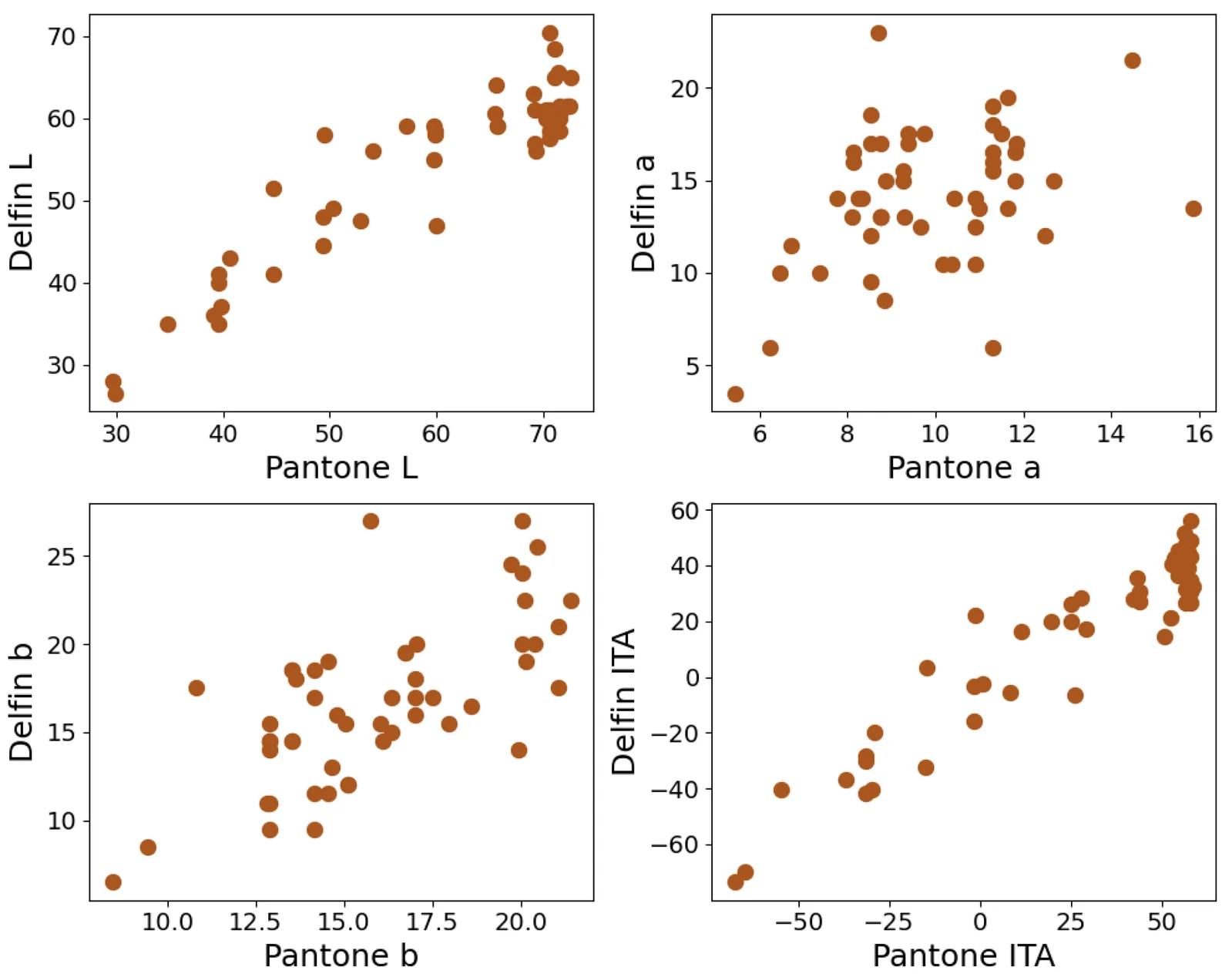
Comparison of L*a*b and ITA values from Pantone vs. Delfin.

We compared ITA and components of $L^{*}a^{*}b^{*}$ from Pantone and Delfin in Figure 4. We observed statistically significant correlation in luminance (L*, r = 0.92, $p_{r}$ = 1.76 × 10^−22^) and ITA (r = 0.95, $p_{r}$ = 2.96 × 10^−26^) values. The correlations in *b* representing the blue to yellow range (r = 0.68, $p_{r}$ = 2.31 × 10^−8^) and *a* representing the red to green range (r = 0.38, $p_{r}$ = 0.005) were relatively weaker, showing the disagreement between the two approaches in measuring the redness and yellowness of the skin.

### 4.5. Comparison of Delfin and Nix Measures

**Figure 5:**
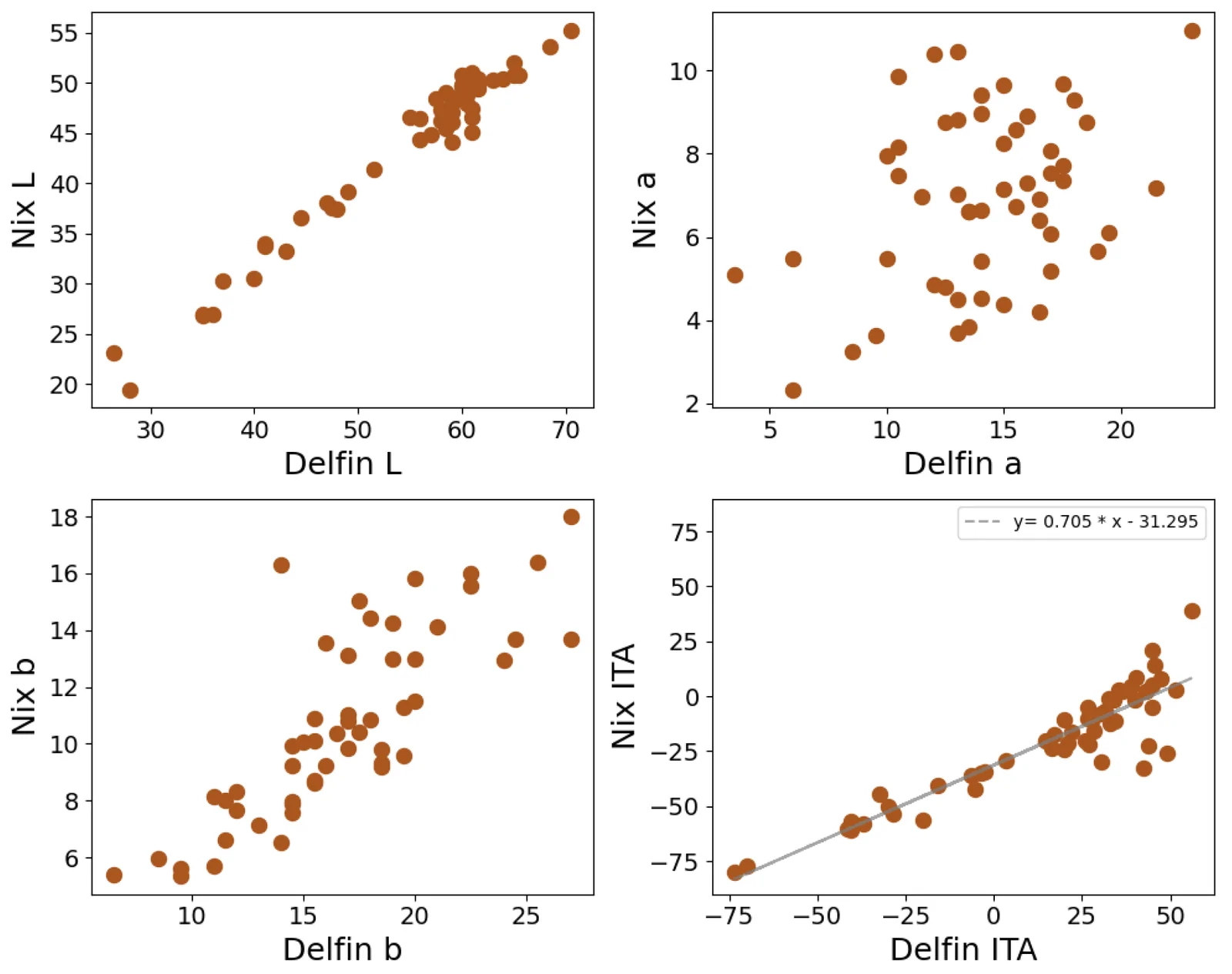
Comparison of L*a*b and ITA values from Delfin and Nix.

Similarly, we pairwise compared between Delfin and Nix-derived ITAs and components of $L^{*}a^{*}b^{*}$ in Figure 5. We observed statistically strong correlations (r = 0.98, $p_{r}$ = 2.11 × 10^−39^) in luminance (L*) and ITA (r = 0.91, $p_{r}$ = 7.54 × 10^−21^), moderate agreement in the *b* component (r = 0.81, $p_{r}$ = 3.10 × 10^−13^) and weaker agreement in the *a* component (r = 0.36, $p_{r}$ = 0.008).

Linear regression on Nix-derived ITA versus Delfin ITA yielded a slope of 0.71 and a y-intersect at −31.3°, which was consistent with Figure 2 where the Nix-derived ITA appeared to be offset towards lower values compared to Delfin ITA.

### 4.6. Discrepancies Between Pantone, Delfin, and Nix

**Figure 6:**
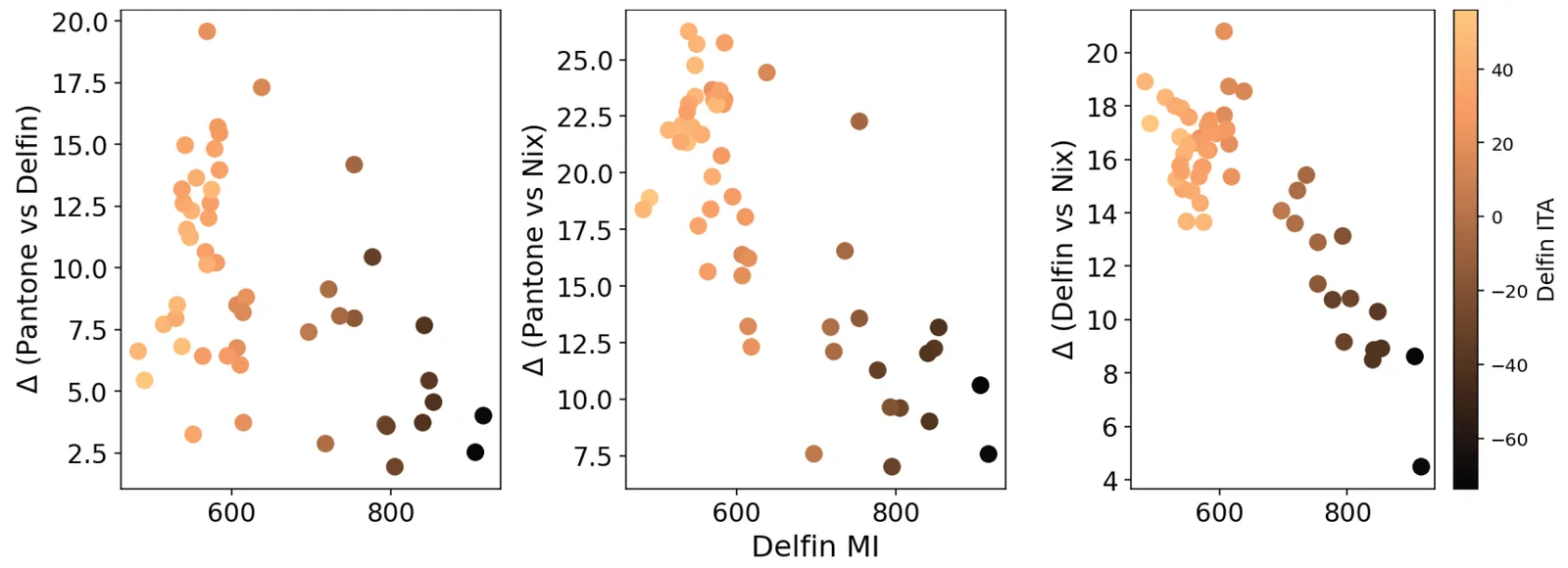
$\Delta^{*}$ value dependence on skin MI.

**Figure 7:**
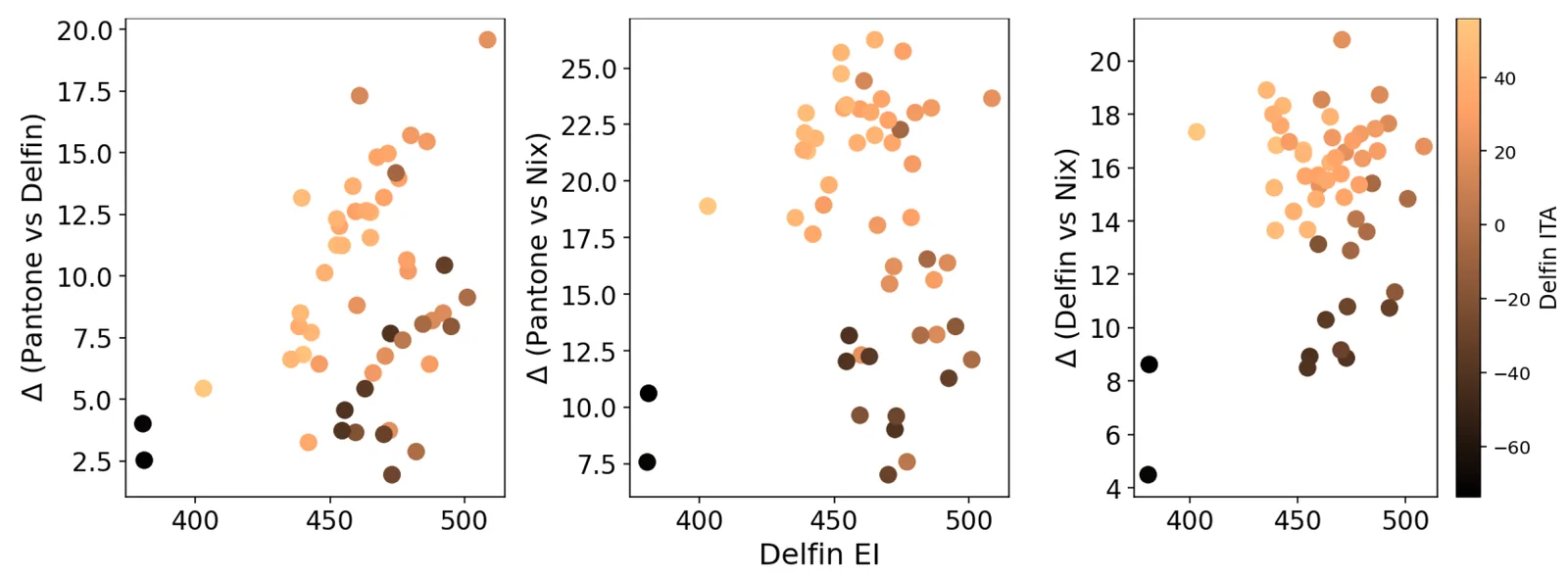
$\Delta^{*}$ value dependence on skin EI.

To measure the difference in skin hue as captured by Delfin, Nix, and Pantone, we computed pairwise $\Delta^{*}$ (2). These were compared against MI and EI reported by Delfin, and were plotted in Figure 6 and Figure 7, respectively. It can be seen on the y-axes that the $\Delta^{*}$ values between Pantone and Delfin ranged 9.17 ± 4.24, Pantone and Nix ranged 18.05 ± 5.56, and Delfin and Nix ranged 14.88 ± 3.30. Statistical correlations are reported in Table 3. There were statistically significant negative correlations between all $\Delta^{*}$ and MI, and statistically significant positive correlations between $\Delta^{*}$ and Delfin ITA. These results demonstrate the higher disagreements between different skin tone measurement approaches for participants with lighter skin tones. The disagreement between the approaches were less influenced by the skin redness as shown by the lower statistical significance in the correlations between $\Delta^{*}$ and EI ($p_{r}$ ranged from 0.01 to 0.85).

## 5. Discussion

In this study we analyzed the skin tone measurements from 52 participants using multiple methods. While the methods demonstrated agreement in terms of linear correlation, and the ICC values indicated good measurement repeatability, significant differences in ITA values were observed across measurement methods. These differences were large enough to influence skin tone classifications, as defined in [13,25] and as visualized in Figure 2, as well as in [18]. When considering $L^{*}a^{*}b^{*}$, the *a* and *b* components generally correlated between methods, but not as strongly as L* and ITA, suggesting potential discrepancies in the reporting of redness and yellowness. Interestingly, differences in $L^{*}a^{*}b^{*}$ (represented by $\Delta^{*}$) were higher for individuals with lighter skin tone. These unexpected findings have implications for both future research and clinical applications, and are further discussed in the following subsections.

### 5.1. Effect of Method on Reported Skin Tone

Our results indicate that the choice of skin tone estimation method affects the ITA measurements (Figure 2). Overall, there was strong correlation between the self-reported FST measurement and the other measures (Figure 3). However, subjective measurements (whether using the FST or the Pantone palette) tended to yield lighter skin tone classifications compared to device-based measurements. Among all methods, the Pantone palette method yielded the lightest reported skin tones, and the Nix device reported the darkest skin tones (Figure 2). Participants and observers reported that it was challenging to select among similar color swatches in the Pantone palette, as many shades appeared perceptually indistinguishable. This may partially explain some of the discrepancy observed in measurements based on Pantone. This is also consistent with the findings reported by Gordon et al. [26].

On average, the Nix-derived ITA values were observed as 35.73° lower than those reported by the Delfin. In the most extreme case, a participant self-reported having very light skin (FST I), while the Nix reported an ITA of −32° corresponding to FST VI. Similar outcomes were also observed in other studies. For example, Gordon et al. [26] reported darker readings with Nix when compared with an industry standard device, although they did not quantify the discrepancy in terms of ITA. Gornitsky et al. [1] also described instances where two visibly lighter skinned participants yielded similar Nix readings that, once converted $L^{*}a^{*}b^{*}$ values to ITA, corresponded to dark skin tones.

Discrepancy between ITA measurements obtained from different methods were statistically significantly greater for lighter skin tones (Table 2). This pattern is also evident in Figure 2, and appeared to be substantiated by the inconsistencies in $L^{*}a^{*}b^{*}$, as shown by correlations involving $\Delta^{*}$, Delfin ITA, and MI (Table 3, Figure 6). More specifically, higher $\Delta^{*}$ values were associated with lower MI and higher ITA values, indicating greater variability among lighter-skinned individuals.

Discrepancies were also present among participants with darker skin tones. Even small discrepancies can negatively affect sampling diversity [18], for example by overestimating and misrepresenting the true number of participants with darker skin tones. Recent FDA draft guidelines for oximeter testing explicitly require that at least 12.5% of participants have very dark skin—defined by an ITA below −50° [23]. In our dataset, the number of participants meeting this criterion varies depending on the measurement method used, ranging from 2 based on the Delfin, to 9 using the Nix (see Table 4). Although no gold standard currently exists for skin tone measurement, it nonetheless seems prudent to validate the output of devices to ensure that reported dark skin tones are, in fact, dark skin tones. Consensus between methods or devices from different manufacturers may be one way to carry out such a validation. Failure to verify measurements accuracy risks producing poor quality data and inequitable negative outcomes.

### 5.2. Effect of Method on Reported Skin Hue

Additional nuance can be captured by analyzing all components in $L^{*}a^{*}b^{*}$. Because the ITA is a mixture of the *L* and *b* values, similar correlations involving these parameters are ITA are expected. When considering the results and statistical correlations presented in Figure 4 and Figure 5, we observed strong agreement between methods on the lightness (*L*) component, and weaker agreement on the yellowness (*b*) and redness (*a*) components. In our study, these discrepancies were stronger in individuals with lighter skin as observed in the correlations between the Delfin ITA and $\Delta^{*}$ in Table 3 and Figure 6. If skin hue is important factor for research or clinical purposes, it is important to recognize that different measurement methods may not yield comparable results. As with overall skin tone, validation of the results between methods or devices by different manufacturers is recommended.

### 5.3. Cost and Usability of Colorimeters

While laboratory-grade spectrophotometers and colorimeters are widely regarded as the reference standard for objective skin color measurement due to their high accuracy and repeatability, their relatively high cost may limit their use in routine clinical practice, large-scale population studies, and resource-limited settings. For example, the price of a Konica Minolta CM700d spectrophotometer which has been used in previous studies [18,22], typically ranges around 10,000 USD. In contrast, the prices of devices evaluated in this study are more affordable, specifically, Nix Spectro 2 spectrophotometer costing only around 1500 USD and Delfin SkinColorCatch colorimeter costing around 8500 USD. While they may exhibit slightly lower accuracies compared to high-end laboratory instruments, the choice of device should depend on the intended application. High-end spectrophotometers remain preferable when maximum measurement precision is required, whereas lower-cost portable devices may represent a more cost-effective solution when accessibility, scalability, and ease of deployment are prioritized.

### 5.4. Limitations of the Study

As indicated in the Methodology, every effort was made to obtain a diverse sample, but participants were recruited from a geographical area with a majority of Caucasian individuals and recruitment was not limited in each skin tone category. As a result, the sample is not equally balanced between different skin tones, limiting the generalizability of the findings across diverse populations. We did not record information on ancestry or ethnicity which might affect how skin tone or color is perceived and measured in subjective techniques, as well as cultural or perceptual biases.

A single unit of each device was used throughout the study. While the manufacturers’ calibration procedures were followed, the performances of the devices might not be representative of all units, and device-specific calibration drift or unit-to-unit variability is not precisely distinguished. The algorithms implemented by the manufacturers may change in the future.

In order to be consistent with previous work in our lab [5], we chose to use FST. However, text-based scales like FST have lower precision and resolution, and are heavily dependent on the reporter’s subjective perception. Several participants indicated that the 6-point scale did not adequately capture their skin tone and instead reported values between whole numbers (for example, 2.5). An alternative scale such as the Monk Skin Tone Scale might be better for identifying and distinguishing skin tones. Future work will include one or more such alternative scales.

## 6. Conclusions

Accurately measuring human skin tone remains a complex research challenge. Skin tone measurement tools used in this study (self-reported FST, observer-reported FST, palette-based skin tone and objective measurements from two commercially-available devices) showed statistically significant correlations with respect to ITA and the luminance (L*) component of the $L^{*}a^{*}b^{*}$ space. Nonetheless, notable discrepancies were observed, particularly in the numeric ITA values from the devices and in hue measurements. Although some existing skin tone tools may be suitable for specific research or clinical applications where absolute accuracy is not critical, our findings underscore the need for further development of validated, reliable, and consistent skin tone measurement methods.

## Figures and Tables

**Table 1:** Number of participants in each skin tone classification defined according to their Delfin ITA measurements [13,25].

Skin Tone Classification	ITA Range	Number of Participants
Very light	55° < ITA < 90°	1
Light	41° < ITA < 55°	10
Intermediate	28° < ITA < 41°	14
Tan	10° < ITA < 28°	11
Brown	−30° < ITA < 10°	8
Dark	−90° < ITA < −30°	8
Total		52

**Table 2:** Mean signed differences of ITA for lighter and darker skin tones.

Skin Tone Classification	Pantone & Nix	Delfin & Nix	Pantone & Delfin
FST I–III	56.49	42.41	14.07
FST IV–VI	37.06	29.53	7.52
$p_{u}$	8.03 × 10^−4^	1.35 × 10^−3^	0.05

**Table 3:** Correlation between $\Delta^{*}$ with skin MI, EI, and Delfin ITA.

	$\Delta^{*}$ Pantone Delfin	$\Delta^{*}$ Pantone Nix	$\Delta^{*}$ Delfin Nix
Skin MI	r = −0.49,	r = −0.77,	r = −0.85,
	$p_{r}$ = 0.0002	$p_{r}$ = 9.9 × 10^−12^	$p_{r}$ = 1.34 × 10^−15^
Skin EI	r = 0.35,	r = 0.02,	r = 0.24,
	$p_{r}$ = 0.01	$p_{r}$ = 0.85	$p_{r}$ = 0.07
Delfin ITA	r = 0.48,	r = 0.78,	r = 0.83,
	$p_{r}$ = 0.0003	$p_{r}$ = 3.45 × 10^−12^	$p_{r}$ = 2.08 × 10^−14^

**Table 4:** Number of participants with extreme readings for each sampling method.

Color Sampling Method	*n* ITA >55°	*n* ITA <−50°
Delfin	1	2
Nix	0	9
Pantone scale	17	3
FST scale	*n* cat. 1	*n* cat. 6
observer-reported	3	5
self-reported	8	5

## Data Availability

The raw data supporting the conclusions of this article will be made available by the authors on request.

## Abbreviations

The following abbreviations are used in this manuscript:

ITA	Individual Typology Angle
FST	Fitzpatrick Skin Type
MST	Monk Skin Tone
MI	Melanin Index
EI	Erythema Index
ICC	Intraclass Correlation Coefficient
